# CNS vasculitis and stroke as a complication of DOCK8 deficiency: a case report

**DOI:** 10.1186/s12883-016-0578-3

**Published:** 2016-04-26

**Authors:** Suzan A. AlKhater

**Affiliations:** Department of Pediatrics, College of Medicine, University of Dammam, Dammam, Saudi Arabia; King Fahad University Hospital, P.O. Box 2208, Al-Khobar, 31592 Saudi Arabia

**Keywords:** DOCK8 deficiency, Hyperimmunity, Hyperimmunoglobulin E syndrome, Moyamoya, Mycophenolate mofetil, Stroke, Vasculitis

## Abstract

**Background:**

Primary immunodeficiency disorders associated with autoimmunity are poorly understood. Central nervous system (CNS) vasculitis can complicate the courses of such entities, but it is underappreciated. Deletion of the *dedicator of cytokinesis 8* (*DOCK8*) gene is considered to be the autosomal recessive form of hyperimmunoglobulin E syndrome which is a rare type of primary immunodeficiency disease characterized by elevated levels of IgE antibody, eczema, and recurrent staphylococcal infections. DOCK8 deletion is associated with fatal CNS vasculitis. However, descriptions of such cases and their outcomes are scarce in the literature.

**Case presentation:**

This report describes a young female with a *DOCK8* gene deletion presenting acutely with squint, fatigue and visual hallucinations. The patient was diagnosed as having neuritis of the third oculomotor nerve and encephalitis, which were thought to be related to her underlying immune deficiency, however, she subsequently was diagnosed with CNS vasculitis based on brain magnetic imaging and magnetic resonance angiography findings. We provide here a comprehensive description of the patient’s clinical outcome and outline an effective treatment approach that may be useful for similar patients and includes the use of steroids and mycophenolate mofetil (MMF). The treatment was well tolerated and enabled the patient to recover most of her neurological deficits. However, despite the initial improvement, she later developed stroke.

**Conclusions:**

To the best of our knowledge, this is the first report in the literature of a case of primary immunodeficiency complicated by CNS vasculitis demonstrating a successful outcome. Our observations indicate that the combination of MMF and steroids is an effective treatment for CNS vasculitis associated with DOCK8 deficiency. However, lack of awareness of the neurological comorbidities associated with primary immunodeficiencies and the delay in diagnosis likely contributed to the development of acute cerebral infarction. Early treatment and aggressive control of the disease’s initial inflammation is essential for preventing catastrophic stroke.

## Background

Primary immunodeficiency disorders are genetic diseases that result in a defective immune system [1]. Affected individuals are predisposed to severe infections complicated by the coexistence of hyperimmunity, which results in various autoimmune and inflammatory disorders [2–4]. Hyperimmunoglobulin E syndrome (HIES) is a type of primary immunodeficiency characterized by elevated levels of IgE antibody, eczema, and recurrent staphylococcal infections [5, 6]. Two disease entities have been identified: an autosomal dominant form caused by a STAT3 gene mutation (OMIM#102582) [7] and an autosomal recessive form primarily caused by a loss-of-function mutation in the dedicator of cytokinesis 8 (DOCK8) gene (OMIM#611432) [8, 9]. The latter condition was identified as a distinct disease entity even before its diagnosis could be confirmed by genetic testing [10] and is characterized by persistent viral skin infections and mucocutaneous candidiasis [8, 9]. A mutation in the tyrosine kinase 2 gene (OMIM#176941) has also been described, but only in one patient who had an unusual susceptibility to viral and mycobacterial infections [11].

HIES is associated with a wide spectrum of vascular abnormalities [12, 13]. The mechanism by which DOCK8 deficiency, in particular, affects patients appears to be related to underlying autoimmunity caused by partial T cell deficiency, resulting in dysregulation and leading to elevated risks of developing both systemic and central nervous system (CNS) vasculitis [14, 15]. The role of hypereosinophilia in the development of vascular anomalies has yet to be investigated [15]. Despite this association, cases of vasculitis and CNS involvement associated with *DOCK8* mutations have been reported only sporadically in the literature [8, 16], which is a major drawback with respect to the development of effective therapies for these vasculitides. No guidelines are available for the treatment of patients with CNS vasculitis complicating a primary immunodeficiency disorder. Currently, the most accepted approach for treating such patients is to adapt an immunosuppressive protocol developed by Hutchinson et al. [17] and designed to treat patients with primary CNS vasculitis. However, said protocol may be inappropriate for patients with an underlying immunodeficiency.

In this report, we describe the challenges of treating a patient with CNS vasculitis in the context of immunodeficiency associated with a *DOCK8* gene deletion.

## Case presentation

A Yemeni female born to consanguineous parents presented at the age of 6 years with eczema, recurrent bronchopneumonia, and skin abscesses. Additionally, she exhibited persistent Molluscum contagiosum infections of the skin and recurrences of Herpes zoster involving multiple dermatomes.

**Fig. 1:**
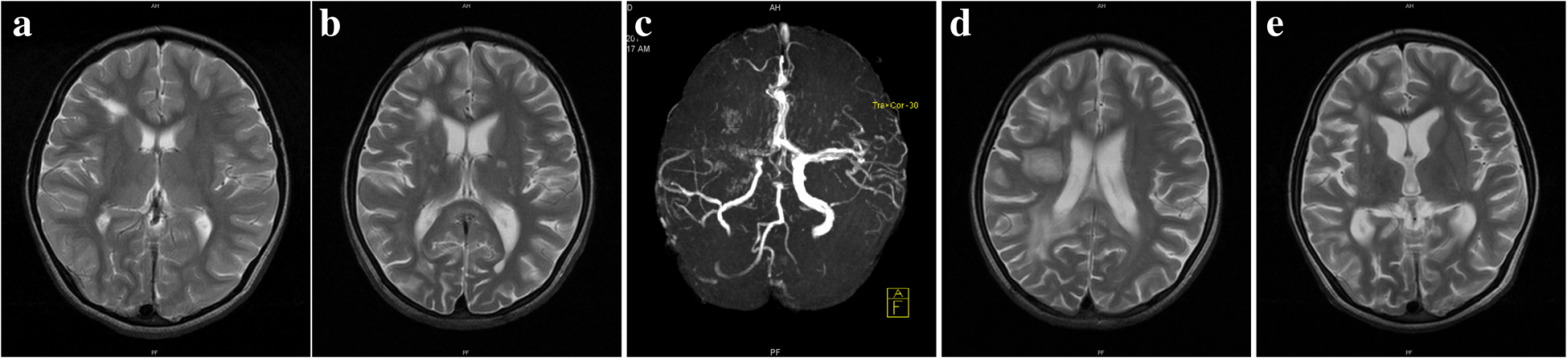
The brain MRI findings of a patient affected by CNS vasculitis and stroke. **a** A baseline MRI image showing T2 hyperintensities involving the right frontal white matter and basal ganglia. **b** Analysis performed following the diagnosis of CNS vasculitis, demonstrating multiple high signal intensity lesions in the periventricular deep and subcortical white matter. **c** The characteristic appearance of moyamoya phenomena-induced vasculopathy is noted. **d** Nine days later, following the development of stroke symptoms, additional changes consistent with white matter demyelination were identified. **e** Three months later, there were improvements in the high signal intensity lesions and no recurrences of infarction

Immunological analysis revealed leukocytosis at 31 × 10^9^/L (4.0–12.0), extreme peripheral eosinophilia as high as 18,000 × 10^9^/L (0–0.5), an elevated serum IgE level of 41,000 IU/ml (0–52 IU/ml), and an elevated IgG level of 2,530 mg/dl (400–1,600 mg/dl). Magnetic resonance imaging (MRI) demonstrated the presence of right frontal white matter and basal ganglia hyperintensities consistent with previous infarcts (Fig. 1a). Based on her clinical presentation, she was evaluated for *DOCK8* gene deletion. Sequencing of the *DOCK8* gene by polymerase chain reaction (PCR) and multiplex ligation probe amplification (MLPA), performed as previously described [9], revealed the presence of a large homozygous deletion of the gene on chromosome 9, consistent with the diagnosis of *DOCK8* gene deletion. The patient’s parents were heterozygous and a younger brother was homozygous for this mutation. The patient’s initial treatment consisted of prophylactic medications, including trimethoprim/sulfamethoxazole (co-trimoxazole), itraconazole, and acyclovir. A monthly intravenous immunoglobulin infusion was initiated at a dosage of 500 mg/kg body weight. However, she had not been compliant with her monthly infusions.

**Fig. 2:**
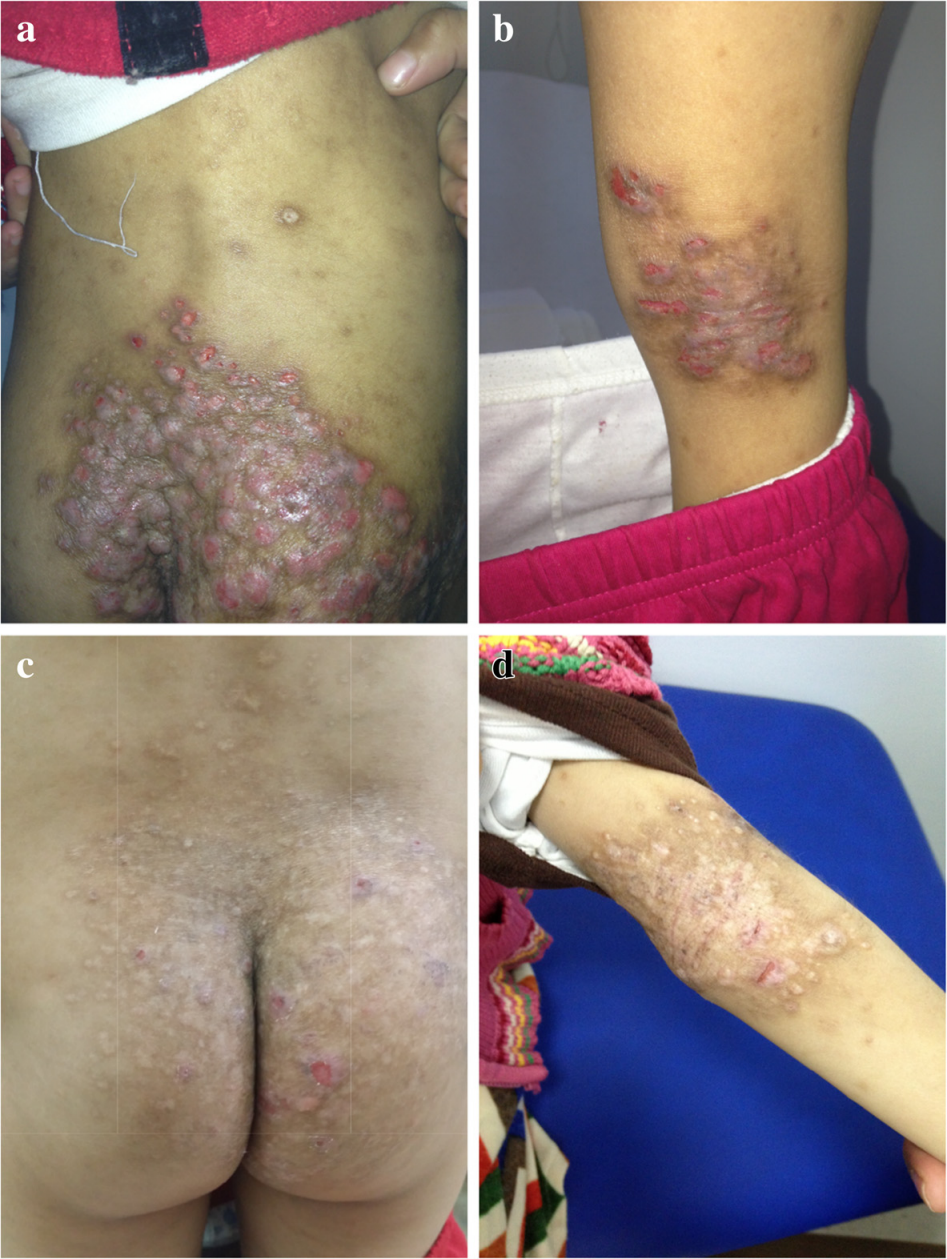
Representative pictures of the dermatitis and eczematous lesions on the back, buttocks and flexures of the patient before therapy (**a**, **b**) and 12 months into steroid and MMF therapy (**c**, **d**)

The patient was stable for two years prior to her current presentation at the age of 8 years, when she was found to have dysphasia and visual hallucinations lasting for 3 weeks, as well as acute left eye squinting, ipsilateral ptosis, and fatigue. Her last immunoglobulin infusion was administered 2 months prior. During this 3-week period, the patient had sought advice from multiple services, including the neurology and ophthalmology outpatient services. She was diagnosed with neuritis of the third oculomotor nerve, which was thought to be caused by the inflammatory nature of her underlying disease and was managed conservatively. The patient continued to be unwell and further developed dizziness, unsteady gait, and excessive sleepiness, for which she was brought to the emergency room and was admitted with a presumptive diagnosis of encephalitis. Upon admission, the patient was physically examined and was noted to have an ill appearance, with an oral temperature of 38.4 °C. Her skin had no active herpetic or vasculitic lesions but showed signs of severe dermatitis on the back and buttocks and eczematous eruptions on the flexures of the arms and popliteal region (Fig. 2a and b). Her neurological examination was significant for left oculomotor nerve and left upper motor neuron facial palsy. Upper and lower limb muscle power were each 4/5, with bilaterally exaggerated tone and reflexes.

Laboratory tests showed a white blood cell count of 16.6 × 10^9^/L, a hemoglobin level of 10.4 g/dl, and a platelet count of 875 × 10^9^/L. The erythrocyte sedimentation rate was 25 mm/h. Her cerebrospinal fluid was colorless, with no cells, normal glucose and protein levels and a negative gram stain. Extensive microbiological testing of the cerebrospinal fluid and blood was performed and yielded negative results (Table 1). An autoimmune diagnostic work-up yielded a high antinuclear antibody titer (1:640) with a speckled immunofluorescence pattern; however, the results were negative for antineutrophil cytoplasmic antibody, anticardiolipin antibody, antiphospholipid antibody, and lupus anticoagulant. The patient’s complement levels, protein C and S levels, and coagulation profiles were normal. Electroencephalography revealed no epileptiform discharges. MRI demonstrated an increase in white matter periventricular high signal intensity lesions compared with previous images (Fig. 1b). Diffusion-weighted imaging (DWI) revealed diffusion restriction characteristic of acute ischemia (not shown). Magnetic resonance angiography (MRA) indicated narrowing and occlusion of the right middle cerebral and internal carotid arteries, with multiple collateral vessels giving the appearance of a “puff of smoke”, features consistent with moyamoya vasculopathy (Fig. 1c). These findings were suggestive of a vascular insult characteristic of vasculitis. No brain biopsy or conventional cerebral angiography was performed because of the lack of test facilities.

**Table 1:** Laboratory investigation

	Patient result	Patient result
Cerebrospinal fluid	Appearance	Clear
(CSF)	Cells	0
	Protein (mg/dl)	25
	Glucose (mg/dl)	80
	Glucose (CSF:serum)	0.8
	Bacterial culture	Negative
	Fungal culture	Negative
	*Mycobacterium tuberculosis*	Negative
	*Cryptococcus neoformans*	Negative
	Herpes simplex virus 1 & 2	Negative
	Enterovirus	Negative
	Polyomavirus JC (JCV)	Negative
	West Nile virus	Negative
Blood serology	Herpes virus 1 & 2	Negative
	Varicella	Negative
	Mycoplasma pneumonia	Negative
	CMV, EBV	Negative
	Mumps, measles	Negative
	Parvovirus B19	Negative
	HIV	Negative

The patient was administered combined immunosuppressive therapy. The treatment consisted of an induction phase in which pulses of steroids were administered at a dose of 30 mg/kg/day (maximum of 1 g/day) for 5 days, followed by a maintenance phase in which 2 mg/kg/day (maximum of 60 mg/day) of oral steroids was administered. Mycophenolate mofetil (MMF) was initiated at a dose of 800 mg/m^2^/day in two divided doses and continued throughout the maintenance phase. This therapeutic approach was modified from that described in the international childhood CNS vasculitis outcome study [17]. Antimicrobial agents, including ceftriaxone, vancomycin and acyclovir, were administered for the first 72 h but were discontinued after ruling out infectious agents.

The patient made remarkable progress and was able to regain full consciousness and activity by the third day of the steroid-pulse phase. A cranial nerve examination demonstrated gradual improvement in her neurological deficits. However, at 1 week following the initiation of immunosuppressive therapy, the patient developed aphasia and left-sided hemiparesis that more greatly affected the faciobrachial region and spared the lower extremity. A repeat MRI demonstrated more extensive white matter changes suggestive of an acute cortical infarct (Fig. 1d), and DWI identified marginal restrictions in the parietal region and blooming artifacts in the parietal periventricular region, findings suggestive of secondary hemorrhagic changes (not shown).

**Fig. 3:**
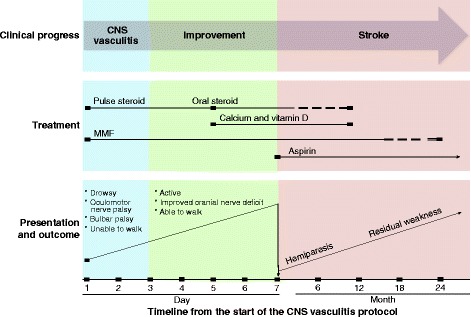
Overview of the progress and therapeutic response of a patient with DOCK8 deletion and CNS vasculitis. Note: The solid line indicates full doses of the medications whereas the dashed line indicates tapered doses

Based on this deterioration, acetylsalicylic acid was added to the patient’s regimen at an initial dose of 5 mg/kg, followed by a reduced dose of 3 mg/kg, which profoundly improved her condition. During the next several months, her neurological deficits improved, and she regained most of her neurological function except for some residual distal weakness. A follow-up MRI performed 3 months following this vascular event revealed improvements in the high-signal lesions and no new lesions or either acute or sub-acute infarcts (Fig. 1e). Steroid therapy was gradually tapered every 4 weeks (60-50-40-30-25-20-17.5-15-12.5-10-7.5-5-2.5-0 mg/day) and was successfully stopped over a 12 month period. The patient received vitamin D and calcium supplementation while she was on steroids. She continued on the combined MMF and acetylsalicylic acid therapy for 18 months, after which she was completely weaned from MMF over another 6-month period. Prophylactic aspirin at a dose of 1 mg/kg/day was continued for the prevention of possible future cerebrovascular events. We monitored the patient for 2 years following the vasculitis and stroke events. She remains symptom-free, and no vasculitis or infectious flare-ups have occurred. In fact, she has shown remarkable improvements in her eczema and skin dermatitis lesions (Fig. 2c and d). The clinical progression of the patient while on the CNS vasculitis protocol is illustrated in Fig. 3.

## Discussion

Loss-of-function mutations in the *DOCK8* gene cause a rare form of primary immunodeficiency [8, 9]. Patients carrying these mutations have a unique hyperimmune system that predisposes them to a wide spectrum of maladies [14, 15]. Various vascular anomalies have been reported in both forms of HIES, including aneurysms, vascular ectasia, and vascular thrombotic events [12]. However, CNS vasculitis [8] and moyamoya [16] are most frequently encountered in patients with DOCK8 deficiency. Brain abnormalities have also been frequently reported in these patients and manifest as MRI hyperintensities [13]. These hyperintensities were observed in our patient and were attributed to old infarcts.

The literature lacks sufficient reports on CNS vasculitis associated with immunodeficiency disorders. Additionally, there is no definitive treatment protocol for such patients. Therefore, the development of new standardized therapeutic approaches for the management of this condition is imperative. In this report, we described the case of a young female affected by CNS vasculitis associated with DOCK8 deficiency and described her responses to MMF and corticosteroid therapy. This is the first report to describe the clinical presentation and outcome of a patient with primary immunodeficiency complicated by CNS vasculitis treated with specific immunosuppressive agents.

Managing patients with CNS vasculitis in the context of a defective immune system is extremely challenging. Clinicians are confronted by the need to aggressively immunosuppress such patients. However, this approach may result in further suppression of an already weakened immune system. Therefore, careful consideration is necessary when selecting an immune suppressant. Immunosuppressive therapy protocols for childhood primary vasculitis have been published previously [17]. The immunosuppressants employed included intravenous cyclophosphamide and steroids during the induction phase, followed by maintenance therapy with either MMF or azathioprine and a subsequent steroid taper [17]. In contrast, we started our patient on MMF for both the induction and maintenance phases.

MMF is a prodrug that is well known for its immunosuppressive effects, which are mainly exerted through the inhibition of inosine monophosphate dehydrogenase, a rate-limiting enzyme involved in the *de novo* synthesis of purine nucleotides [18]. As lymphocytes are highly dependent on this enzyme for their proliferation, MMF is considered a lymphocyte-selective agent that causes inhibition of lymphocytes proliferation, immune suppression, and inhibition of antibody production [18]. The potent anti-inflammatory activity of this drug is mediated through several mechanisms, including the inhibition of proinflammatory cytokine production and the expression of adhesion molecules, resulting in blocking of the recruitment of immune cells to the site of inflammation [19]. Interestingly, this anti-inflammatory property is believed to have a neuroprotective effect in ischemic stroke, as has been demonstrated in experimental models [19, 20]. In one report, the use of MMF in stroke-affected rat models resulted in decreases in the signal intensities and volumes of infarcts observed on DWI and an increase in the motor performance of the animals [19].

MMF was selected for the treatment of our patient for both induction and maintenance in an attempt to reduce the risk of infection, given the compound's relatively mild immunosuppressive activity compared with cyclophosphamide and its better safety profile [21]. The patient tolerated the compound well and exhibited no significant adverse effects related to her therapy, with the exception of hypokalemia, which was managed with oral potassium supplementation. To date, our patient has not experienced any significant illnesses, including flare-ups of her skin infections, and presented a noticeable improvement in her skin condition while receiving this therapy. Most importantly, the treatment was effective in reversing most of the patient’s neurological deficits.

One important consideration regarding the management of patients with CNS vasculitis is the potential development of stroke due to vessel inflammation and stenosis [22, 23]. In our patient, stroke occurred later, following an initial improvement. Early recognition of the neurological signs of CNS vasculitis and the prompt treatment of this condition, which should include aggressive management of the disease's initial inflammation, would allow for increased control over the disease's progression. We started our patient on 800 mg/m^2^/day of MMF; however, the immunosuppressive effect could have been optimized by increasing the dosage of the drug as the therapeutic dose ranges from from 800–1,200 mg/m^2^/day.

Furthermore, our patient was started on low-dose aspirin as an anticoagulation therapy for the management of her stroke. Aspirin at a low dose has both antiplatelet and anti-inflammatory properties that are mediated by an increased level of plasma nitric oxide [24] and inhibition of platelet COX-1-dependent thromboxane formation [25]. Inflammation and thrombosis play key roles in the development of CNS vasculitis and stroke [23]. Aspirin has been considered a standard therapy in children with stroke [26], however, its use in the treatment of CNS vasculitis or for the prevention of stroke in such patients has not been studied.

Additionally, moyamoya, a steno-occlusive cerebral angiopathy resulting in vascular collateral formation [27], was noted in our patient. DOCK8-deficient patients reportedly develop moyamoya [16], which is considered a consequence of ischemia. However, recent reports have suggested the presence of an underlying autoimmune disease process related to T cell dysregulation in these patients, particularly in patients with unilateral disease, as in our patient [28]. Knowledge of medical management and revascularization procedures for patients similar to ours is unfortunately lacking. Notably, caution should be exercised when prescribing antiplatelet therapy to affected patients, as hemorrhage is a known complication of moyamoya vasculopathies, especially the hemorrhagic type [29]. Fortunately, the latter has rarely been reported in children [29].

Our goal in reporting this case is to increase awareness of primary immunodeficiency disorders and knowledge regarding their natural histories and comorbidities. The delay in recognizing the neurological comorbidity related to DOCK8 deficiency in our patient likely contributed to the occurrence of catastrophic stroke. We also emphasize the importance of periodic screening for vascular events even in asymptomatic patients, in addition to family counseling about neurological symptoms. Moreover, by reporting this case, we hope to outline a therapeutic approach to treating CNS vasculitis that may be used successfully in immunosuppressed individuals. The only definitive cure for immunodeficiency caused by a *DOCK8* gene deletion is hematopoietic stem cell transplantation [30]; however, access to this treatment may be an issue, either because of the unavailability of a cross-matched donor or because of the lack of an advanced immunology center to perform the procedure. Therefore, it is important to develop alternative and specific means of treating life-threatening events. The potential development of cerebrovascular anomalies and their complications in such patients must be considered in the development of future therapeutic approaches.

## Conclusion

In conclusion, we have described both the clinical presentation and the successful outcome of CNS vasculitis treated with MMF and corticosteroids in a young child with DOCK8 deficiency. Our report suggests that using such an approach is both an effective and safe means of treating autoimmunity and vasculitis. Recognition and timely diagnosis, in addition to more aggressive management of the disease’s initial inflammation, may prevent complications.

### Consent

Written informed consent was obtained from the patient's legal parents for publication of this case report and any accompanying images. A copy of the written consent is available for review by the Editor of this journal.

## Abbreviations

CNS: central nervous system
DOCK8: dedicator of cytokinesis 8
DWI: diffusion-weighted imaging
HIES: hyperimmunoglobulin E syndrome
MLPA: multiplex ligation probe amplification
MMF: mycophenolate mofetil
MRA: magnetic resonance angiogram
MRI: magnetic resonance imaging
PCR: polymerase chain reaction
